# Protocol: does sodium nitrite administration reduce ischaemia-reperfusion injury in patients presenting with acute ST segment elevation myocardial infarction? Nitrites in acute myocardial infarction (NIAMI)

**DOI:** 10.1186/1479-5876-11-116

**Published:** 2013-05-06

**Authors:** Nishat Siddiqi, Margaret Bruce, Christopher J Neil, Baljit Jagpal, Graeme Maclennon, Seonaidh C Cotton, Sofia A Papadopoulo, Nicholas Bunce, Pitt Lim, Konstantin Schwarz, Satnam Singh, David Hildick-Smith, John D Horowitz, Melanie Madhani, Nicholas Boon, Juan-Carlos Kaski, Dana Dawson, Michael P Frenneaux

**Affiliations:** 1 Division of Applied Medicine, School of Medicine and Dentistry, University of Aberdeen, Foresterhill, Aberdeen AB25 2ZD, UK; 2 Centre for Healthcare Randomised Trials (CHaRT) Health Services Research Unit, University of Aberdeen, Health Sciences Building, Foresterhill, Aberdeen AB25 2ZD, UK; 3 Cardiovascular Sciences Research Centre, Cranmer Terrace, London SW17 0RE, UK; 4 Cardiology Department Atkinson Morley Wing, St Georges Healthcare NHS Trust, Blackshaw Road, London SW17 0Q, UK; 5 Brighton and Sussex University Hospitals, Cardiology Research Unit, 1 Abbey Road, Brighton BN2 1ES, UK; 6 Basil Hetzel Institute for Translational Health Research, The Queen Elizabeth Hospital, 28 Woodville Road, Woodville South SA 5011, Australia; 7 Centre for Cardiovascular Sciences College of Medical and Dental Sciences, University of Birmingham, Edgbaston, Birmingham B15 2TT, UK; 8 Centre for Cardiovascular Science, The University of Edinburgh, Chancellor's Building, 49 Little France Crescent, Edinburgh EH16 4SU, UK

**Keywords:** Ischaemia-reperfusion-injury, Myocardial infarction, Sodium nitrite, Primary percutaneous coronary intervention, Cardioprotection

## Abstract

**Background:**

Whilst advances in reperfusion therapies have reduced early mortality from acute myocardial infarction, heart failure remains a common complication, and may develop very early or long after the acute event. Reperfusion itself leads to further tissue damage, a process described as ischaemia-reperfusion-injury (IRI), which contributes up to 50% of the final infarct size. In experimental models nitrite administration potently protects against IRI in several organs, including the heart. In the current study we investigate whether intravenous sodium nitrite administration immediately prior to percutaneous coronary intervention (PCI) in patients with acute ST segment elevation myocardial infarction will reduce myocardial infarct size. This is a phase II, randomised, placebo-controlled, double-blinded and multicentre trial.

**Methods and outcomes:**

The aim of this trial is to determine whether a 5 minute systemic injection of sodium nitrite, administered immediately before opening of the infarct related artery, results in significant reduction of IRI in patients with first acute ST elevation myocardial infarction (MI). The primary clinical end point is the difference in infarct size between sodium nitrite and placebo groups measured using cardiovascular magnetic resonance imaging (CMR) performed at 6–8 days following the AMI and corrected for area at risk (AAR) using the endocardial surface area technique. Secondary end points include (i) plasma creatine kinase and Troponin I measured in blood samples taken pre-injection of the study medication and over the following 72 hours; (ii) infarct size at six months; (iii) Infarct size corrected for AAR measured at 6–8 days using T2 weighted triple inversion recovery (T2-W SPAIR or STIR) CMR imaging; (iv) Left ventricular (LV) ejection fraction measured by CMR at 6–8 days and six months following injection of the study medication; and (v) LV end systolic volume index at 6–8 days and six months.

**Funding, ethics and regulatory approvals:**

This study is funded by a grant from the UK Medical Research Council. This protocol is approved by the Scotland A Research Ethics Committee and has also received clinical trial authorisation from the Medicines and Healthcare products Regulatory Agency (MHRA) (EudraCT number: 2010-023571-26).

**Trial registration:**

ClinicalTrials.gov: NCT01388504 and Current Controlled Trials: ISRCTN57596739

## Background

There are approximately 125,000 acute myocardial infarctions (AMI) in the UK per year (BHF statistics 2008 (http://www.heartstats.org)). Whilst advances in reperfusion therapies such as primary percutaneous coronary angiography (PPCI) have reduced early mortality from AMI [1] morbidity, most commonly resulting from heart failure, which may occur early or long after the MI, remains high [2]. Paradoxically, the act of reperfusion leads to further tissue damage, a process described as ischaemia-reperfusion-injury (IRI) which can account for up to 50% of the final infarct size [3]. Given that infarct size is determined not only by ischaemia but also by IRI, targeting the latter offers the potential to limit myocardial injury and reduce mortality and morbidity.

### Ischaemia reperfusion injury and conditioning

Following reperfusion of an occluded artery, a cascade of events leads ultimately to the opening of the mitochondrial transition pore (MTP) in the inner mitochondrial membrane. Factors promoting opening of the MTP include an increase in intracellular calcium that occurs during ischaemia and increased generation of reactive oxygen species (ROS) and toxic aldehydes [4]. The opening of the MTP leads to irreversible cell death. This results from both acute energetic impairment (due to loss of the electrochemical gradient across the mitochondrial membrane that drives ATP synthesis) and release of the mitochondrial contents into the cytosol, including cytochrome C, which activates pro-apoptotic pathways [5]. Crucially, the opening of the MTP does not occur until approximately two to three minutes after reperfusion. This is because the low pH that is present during ischaemia inhibits its opening, and it is not until the pH starts to recover following reperfusion that the raised calcium and ROS induce opening of the MTP.

In 1986 Murry and colleagues [6] made the seminal observation that brief repeated episodes of myocardial ischaemia reduced the magnitude of myocardial injury caused by a subsequent prolonged episode of coronary occlusion, a phenomenon they termed ischaemic preconditioning and which has since been reproduced in well over 1000 papers [7-9]. Several endogenous conditioning pathways confer cardio-protection against IRI including the Reperfusion Injury Salvage Kinase Pathway (RISK). Ultimately these act by inhibiting the opening of the MTP [7,10]. A corollary of the fact that MTP opening is delayed by some minutes after reperfusion is the potential to reduce IRI by interventions delivered either during ischaemia (perconditioning) or at the time of reperfusion (postconditioning). Thus, graded opening of an occluded coronary artery reduces IRI (direct postconditioning). Subsequent studies have shown that brief periods of ischaemia in other organs confer similar protection to the myocardium, a phenomenon called *remote* ischemic myocardial conditioning [11]. ‘Ischaemic conditioning’ may be replicated by using several pharmacological stimuli, e.g. opiates, cyclosporine, erythropoietin, H/Na exchange inhibitors and nitric oxide donors. Both ischaemic and pharmacological cardiac pre-conditioning are mediated at least in large part via the RISK pathway [10].

There is extensive literature on successful pre, per and post conditioning interventions in animal models of AMI, which have resulted in reduction in final infarct size by up to 50%. Unfortunately, in spite of these very promising results in animal models, translation into benefit in human studies has been inconsistent. There may be several reasons for the poor translation into humans: (i) patients have multiple comorbidities that may make the heart more resistant to conditioning (e.g. age, hypertension and diabetes) [12,13]; (ii) prompt reperfusion with PPCI may minimise the potential benefit from conditioning strategies. Indeed, there is evidence from some of the human intervention studies that the benefit is largely confined to patients with larger infarcts, particularly those associated with occlusion of the left anterior descending artery [14]; (iii) it is known that spontaneous opening and closing of the occluded coronary artery (intermittency) is common in acute myocardial infarction [15], potentially replicating direct post conditioning and in this context any additional conditioning intervention may have limited therapeutic impact.

Nevertheless positive studies in humans have been reported. Remote (forearm) ischaemic perconditioning in acute ST elevation MI (STEMI) (administered in the ambulance *en route* to hospital for PPCI) resulted in a reduction in IRI – expressed as an increase in myocardial salvage compared to a placebo intervention. Although this did not translate into a significant reduction in infarct size for the whole group, a reduced infarct size was seen in the subgroup of patients with left anterior descending coronary artery occlusions [14]. Pharmacological postconditioning using cyclosporine administered prior to PPCI in acute STEMI was reported to decrease the area under the curve for the biomarkers creatine kinase (CK) and Troponin (the primary end point) and a reduction in infarct size (assessed by gadolinium late enhancement on cardiovascular magnetic resonance imaging (CMR) performed at five days) was reported in a subgroup of 27 patients [16].

### Nitrite, NO and cardioprotection

Plasma nitrite is derived from oxidation (principally by Caeruloplasmin) of endothelially derived nitric oxide (NO), and by the reduction of dietary inorganic nitrate by bacteria in the salivary glands and gastro-intestinal tract [17]. Under normoxic conditions, nitrite has a relatively modest vasorelaxant effect compared to organic nitrates. However in vascular rings highly acidic solutions of nitrite causes marked vasorelaxation [18], largely due to reduction to NO by acid disproportionation. Under less extreme conditions modest hypoxia and/or acidosis results in reduction of nitrite to NO by various mechanisms including by mitochondrial aldehyde dehydrogenase type 2 (ALDH2) [19], cytochrome C [20], deoxygenated forms of heme proteins including haemoglobin and myoglobin [21] endothelial nitric oxide synthase, and xanthine oxidoreductase [22]. Consequently nitrite induced vasodilation is potentiated by hypoxia in vivo [23].

In addition to these vascular effects a number of studies in animal models have now shown that sodium nitrite administered acts as both a pre and a per-conditioning agent [24]. This effect appears to be at least in part due to reduction of nitrite to NO by deoxymyoglobin in the heart, and can be abolished by NO scavengers [25,26]. Low dose nitrite given at the time of commencing resuscitation in a mouse model of cardiac arrest improved both cardiac and neurological function and increased survival [27].

In a study of acute MI in a canine model, an infusion of sodium nitrite administered during coronary occlusion (i.e. a perconditioning regime) conferred significant cardioprotection. Pertinently, there was no significant difference in cardioprotection between a group that received an infusion of sodium nitrite during the final five minutes of the two hour coronary occlusion and that seen in a group having an infusion throughout the final 60 minutes of the occlusion [28].

## Trial design and methods

NIAMI is a UK Medical Research Council funded, multi-centre, double blind, placebo-controlled, randomised trial evaluating sodium nitrite versus placebo. The trial will be conducted in cardiac units in three UK hospitals - Aberdeen Royal Infirmary, St George’s Hospital London and Brighton and Sussex University Hospitals NHS Trust and at the Queen Elizabeth Hospital, Adelaide, Australia.

**Figure 1:**
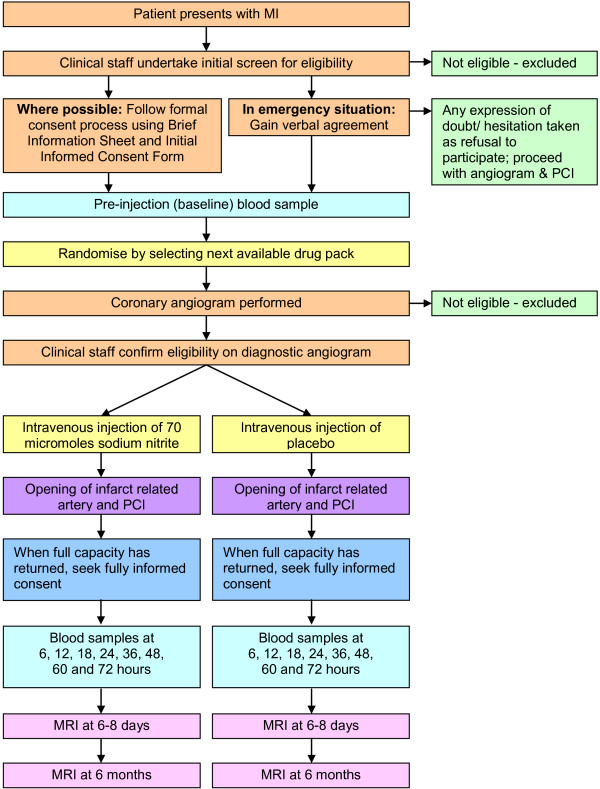
Overview of trial design.

See Figure 1 for an overview of the trial design.

### Trial hypothesis

The hypothesis being tested is: A five minute systemic injection of sodium nitrite, administered immediately before opening of the infarct related artery by PPCI, results in a significant reduction of IRI in patients with first acute STEMI.

### Active treatment

Sterile solution containing 70 micromol sodium nitrite dissolved in 5 ml water injected intravenously over a period of five minutes.

### Placebo

Sterile solution containing 0.9%w/v sodium chloride in 5 ml water injected intravenously over a period of five minutes.

### Selection of participants

As standard practice, clinicians will assess patients presenting with chest pain. Those patients who are potentially eligible will be logged using the inclusion form. Those who are found to be not eligible will have the reason for non-eligibility recorded on the same form.

### Inclusion criteria

Men aged ≥18 years, women aged ≥55 years, and women <55 years who are sterilised, or have had a hysterectomy or have effective contraception and therefore with no possibility of being pregnant;

Presenting within 12 hours of the onset of chest pain and with ECG findings of ST segment elevation of more than 1mm in two contiguous limb leads or 2mm elevation in two contiguous chest leads or new left bundle branch block (LBBB) and for whom the clinical decision has been made to treat with primary PCI;

Patients with posterior infarcts with anterior ST segment depression who meet the other inclusion criteria can also be included;

Occlusion of the culprit related artery (TIMI grade 0 or TIMI grade 1);

Of North European descent.

### Exclusion criteria

Historical or ECG evidence of previous myocardial infarction;

Patients with prior coronary artery bypass grafting (CABG);

Prior revascularisation procedure where this procedure (PCI) was performed in the same territory as the current infarct;

Known or suspected pregnancy;

Contra-indications to CMR;

Patients with cardiac arrest or cardiogenic shock;

Patients with left main coronary occlusion;

Patients with known moderate to severe renal failure (estimated GFR < 30mls/min), or liver failure;

Patients undergoing rescue PCI for failed thrombolysis;

Patients with Left Main stenosis of such severity that after PCI of their culprit lesion (LAD or LCx or RCA) they are likely to require CABG within the time course of the study period (6 months).

### Informed consent

Where possible, formal consent will be obtained prior to including the patient in the trial. The brief information sheet will be provided to patients, and they will be asked to sign an initial consent form if they agree to take part (initial informed consent). However, because this initial consent may not be “fully” informed (either because patients have had opiates to relieve pain or are in a state of considerable anxiety), fully informed consent will be sought from these patients when full capacity has returned. At that stage, patients will receive full written information and an explanation of the study. If they are happy to remain part of the study, they will be asked to sign the consent form. However, in the emergency situation when there is no time to follow the initial informed consent process, and the urgency of providing treatment within the necessary time-window precludes any reasonable attempt to obtain informed consent from a relative or ‘legal representative’, verbal agreement will be sought from the patient by the treating cardiologist by providing a short spoken explanation. In deciding our policy for seeking only verbal agreement, we have taken full account of the provisions in The Medicines for Human Use (Clinical Trials) Amendment (No 2) Regulations 2006, section 31 of Mental Capacity Act (2005) and part 5 of The Adults with Incapacity (Scotland) Act 2000.

In the Australian site, participants are asked to sign an informed consent form prior to inclusion in the study.

### Randomisation

Eligible participants will be randomised to the intervention group or the placebo group on a 1:1 basis. The drug packs (sodium nitrite and placebo) will be manufactured by Tayside Pharmaceuticals. They will be randomised in permuted blocks prior to dispatch to sites. Each drug pack will be allocated a sequential number. At each site, the next available drug pack will be selected and used for eligible patients who give verbal agreement. The number of the drug pack will be recorded on the inclusion form and in the medical records.

### Code break/emergency unblinding

There will be no facility for emergency unblinding. There are two clinical reasons for this decision. Firstly, there is no antidote to the study medication. Secondly, knowledge of the treatment received (sodium nitrite or placebo) would not impact on any management decisions being taken if an adverse event occurs.

### Description and justification of route of administration, dosage, treatment periods

The dose of sodium nitrite is 70 micromoles sodium nitrite dissolved in 5ml water given intravenously over five minutes immediately prior to opening of the infarct related artery. This dose is based on a canine study in which nitrite was infused during myocardial ischaemia, but increased to reflect differences in body weight between this canine model and an average 70kg human [28]. Nitrite has been used for decades as an antidote to cyanide poisoning by inducing methaemoglobinemia [29]. Physiological levels of methaemoglobin within the blood vary from 0-2% [30]. Levels of up to 20% are considered safe in a clinical context [31]. The dose employed in this study is not associated with methaemoglobinaemia [32], although as a precaution patients whose ethnic origin could indicate a high risk of G6PD deficiency will be excluded. This dose has also been shown not to produce significant change in blood pressure in either healthy controls or patients with heart failure (Frenneaux, unpublished data).

### Study end points

#### Primary end point

(i) The difference in infarct size between sodium nitrite and placebo groups measured using CMR performed 6–8 days following the acute myocardial infarction (assessed as extent of late gadolinium enhancement (LGE) and corrected for myocardial area at risk (AAR), which will be assessed by epicardial extension of the LGE area (Endocardial surface area - ESA).

### Secondary end points

(i) Plasma CK and Troponin I area under curve measured in blood samples taken pre-injection of the study medication and at eight time-points over the 72 hours following injection of the study medication;

(ii) CMR measured Infarct size at 6–8 days with AAR measured by T2 weighted triple inversion recovery (T2-W SPAIR or STIR) CMR as a covariate;

(iii) AAR measured at 6–8 days using T2 weighted triple inversion recovery (T2-W SPAIR or STIR) CMR imaging;

(iv) LV ejection fraction measured by CMR at one week and at six months after AMI;

(v) LV end systolic volume index at approximately one week and at six months after AMI;

(vi) Infarct size (CMR) at six months post AMI.

Clinical and safety outcomes will be assessed for the six months following MI (e.g. death, re infarction, further re vascularisation, heart failure, stroke).

### Timing and recording of safety parameters

The number and nature of any serious adverse events in each arm will be recorded. Any serious adverse reactions are likely to occur in the hours following administration of the study drug and not in the following days/weeks. Information regarding safety parameters will be collected until hospital discharge and again at six months. A summary of all serious adverse reactions will be prepared every three months and distributed to the participating investigators, the Co-Sponsors, the manufacturer, the Trial Steering Committee and the Data Monitoring Committee (DMC).

A Developmental Safety Update Report will be prepared annually and submitted to the MHRA Ethics Research Committee in accordance with the guidance on annual safety reporting. The DMC will convene regularly (at least annually) and assess the safety of trial participants and the completeness of data collected. Due to the timing of the primary outcome assessment (see below), it is not anticipated the NIAMI trial be terminated for efficacy or futility. However the DMC may advise that the trial is temporarily or permanently halted based on safety concerns according to the criteria defined in the DMC charter.

### Assessment of end-points

Assessment of the end points will be performed blinded to the treatment.

### Blood samples/testing

#### Collection of samples

Blood samples will be collected pre-injection of the study medication, and at 6, 12, 18, 24, 36, 48, 60 and 72 hours after injection of the study medication.

### Blood analysis

Standard protocols for the analysis of plasma CK (Siemens Advia 2400) and troponin I (Siemens Advia Centaur using the TNI ultra method) will be used. All samples will be tested at the core laboratory at the Aberdeen Royal Infirmary.

### CMR scanning/analysis

Patients found to have contraindications to CMR scanning will not undergo this.

### Scanning process

Scan 1: At 6–8 days post-injection of the study medication:

Gadolinium-enhanced CMR Imaging will be performed to obtain the following measurements for analysis using the parameters below:

•Volumetric LV ejection fraction

•Area at risk (ESA and oedematous area)

•Early assessment of infarct size and extent of microvascular obstruction

•LV end systolic volume index

In addition a clinical CMR report will be produced for the patient’s cardiologist.

Scan 2: At six months post-injection of the study medication: However in those patients in whom further revascularisation or device (pacemaker/ICD) is planned before six months, the second CMR will be brought forward, if necessary to as early as 3–4 months. CMR Imaging will be performed to obtain the following measurements for analysis using the parameters below:

•Late assessment of infarct size by LGE

•LV ejection fraction

•LV end systolic volume index

In addition a clinical CMR report will be produced for the patient’s cardiologist.

### Imaging parameters

The sequences will be standardised between centres as much as possible (given that different vendor equipment is available in one centre) but the individual scan parameters will be adapted according to the individual needs of each patient (for example to match their heart rates). Images will be stored locally following standard clinical practice and transferred to the core lab in Aberdeen for analysis.

### Analysis of images

All images will be analysed off line by a blinded observer in the core lab using software already developed for this purpose. A second blinded observer analysed a sample of 40 images. Analysis of AAR will be performed on the basis of two techniques described below. The first technique relates the length of the subendocardial edge of the gadolinium boundary to the transmural volume of left ventricular myocardium that this underlies (ESA technique) [33]. The rationale of this is that infarction begins in the subendocardial region and extends as a wavefront transmurally; therefore salvage has the effect of limiting this transmural extension. In the second technique the area of oedema is measured using a T2 weighted triple inversion recovery (T2-W SPAIR or STIR) CMR imaging. Oedema develops in the territory supplied by an occluded artery within one hour of occlusion [34]. In 40 NIAMI patients, we demonstrated a median area at risk that was quantatively similar using T2-W oedema and ESA methods but both within and between observer variability was substantially better for ESA (intra-class correlation for ESA was 0.96 and for T2 0.6). Accordingly, we chose to employ ESA for measurement of AAR in the primary end point but retained T2 as an area at risk measurement in the secondary end points.

The infarcted area will be identified from the LGE CMR scan. The infarcted myocardium has conspicuous demarcation on LGE and exquisite contrast compared to the adjacent (nulled) myocardium, therefore tracing the infarct borders poses no problem for analysis. Both the oedematous and infarcted areas will be traced on all short axis slices and the total respective volumes of oedematous/infarcted myocardium calculated in absolute and relative terms to the left ventricular volume using commercially available software (SEGMENT). Volumetric LV ejection fraction will be measured at the initial CMR scan and at six months using standard approaches and commercially available software (CMRTools, Cardiovascular Imaging Solutions, London, UK).

### Statistics

#### Proposed sample size

The primary outcome measure in this study will be the infarct size. We will estimate the difference in infarct size between the treatment and intervention group correcting for area at risk (AAR) and diabetic status using an analysis of covariance framework (ANCOVA) to take advantage of the correlation between IS and AAR to reduce sample size. Data from Cook (personal communication) and the Aarhus study [14] suggest that the AAR in this cohort would be approximately mean 30% (standard deviation 15), and that the IS in the placebo group should be between mean 15% (from Aarhus) to 20% (from Cook) (standard deviation 11). We have chosen the lower of these values because this estimate was from clinical trial data. Data from both these data sets indicate that correlation between AAR and IS is above 0.60. The estimate of treatment effect in the Aarhus study was a reduction of IS from 15% in the control to approximately 9% in the intervention group, a reduction of 6%. This trial proposes more conservative, but still clinically relevant treatment effect size of 4% between the treatment and control group (a standardised effect size of about 0.36).

To explore statistical power and estimate sample size for the trial we used Monte Carlo simulation in Stata 11. (StataCorp. 2009. *Stata Statistical Software: Release 11*. College Station, TX: StataCorp LP.) A model based the above assumptions was used to simulate 10,000 trial data sets for a variety of sample sizes. The simulated data sets were analysed using ANCOVA to reflect the primary trial analysis. The proportion of simulated trials that return p-values less than 0.05 was then counted to evaluate the power of ANCOVA. A sample size of 150 (75 per group) will provide 90% power to detect a difference of 4% between treatment and control group in IS corrected for AAR. The sample size has been expanded by approximately 30-35% to account for loss of outcome measure due to death, those who decline CMR or are unsuitable for CMR due to renal dysfunction and those in whom the CMR scans are technically inadequate. We therefore plan to recruit 200–210 participants.

### Statistical analysis

Data will summarised and reported in adherence with CONSORT guidelines. The analysis of the primary outcome (IS) will use a generalised linear model (ANCOVA), with covariates for area at risk, diabetic status and centre. Only participants that have primary outcome data will be included in the analysis, no attempt will be made to impute data for participants that cannot, for whatever reason, contribute CMR data in the primary analysis. We will test the robustness of results to missing data by conducting sensitivity analyses to suit the pattern of missing data that arises. Secondary analyses will be conducted in a similar manner, using models suitable for the outcome. Blood sample outcomes will be analysed by first deriving an area under the curve and then using a similar strategy to the primary outcome. All estimates of effect will be presented with 95% confidence intervals. All analyses will be done in Stata (StataCorp. 2009. *Stata Statistical Software: Release 11*. College Station, TX: StataCorp LP.)

### Trial oversight committees

#### Data Monitoring Committee (DMC)

The DMC includes three independent members, including an independent statistician. The committee will meet regularly to monitor the unblinded trial data and serious adverse events and make recommendations as to any modifications that are required to be made to the protocol or the termination of all or part of the trial.

### Trial Steering Committee (TSC)

The TSC includes three independent members. The committee will meet regularly to provide oversight for the trial.

### Ethics and regulatory approvals

This protocol and related documents have been reviewed by Scotland A Research Ethics Committee. The study has also received clinical trial authorisation from the Medicines and Healthcare products Regulatory Agency (MHRA) (EudraCT number: 2010-023571-26). Appropriate approvals are in place for the Australian site. The study conforms with the principles of the Declaration of Helsinki and the European Clinical Trials Directive.

### Strengths and weaknesses of this study

This is the first multi-centre, randomised, placebo-controlled and double-blinded trial of sodium nitrite as a conditioning agent in man. We use a robust method of accurately delineating infarct size and area at risk using CMR imaging techniques alongside the standard use of biomarkers. Due to the risk of methaemoglobinaemia in patients at risk of G6PD deficiency, only those of Northern European descent will be recruited. Further studies need to be conducted in other ethnic groups.

## Conclusion

Whilst huge strides have been made to reduce myocardial infarct size by timely opening of the infarct related artery, firstly ischemia and subsequently reperfusion itself cause substantial myocardial injury. Effective therapy to reduce this injury is urgently required if we are to make further progress in limiting infarct size, thereby reducing the subsequent development of heart failure. Whilst soundly based on animal studies, this proposal would be the first in man study to investigate whether intravenous infusion of sodium nitrite reduces myocardial infarct size.

## Competing interests

The authors declared that they have no competing interests.

## Authors’ contributions

All authors listed above fulfil all International Committee of Medical Journal Editors (ICMJE) guidelines for authorship. NS and MPF are the main authors and prepared the manuscript. MPF conceived and substantially contributed to the design of the study. DD, MM, NS, PL, BJ, SC and JCK contributed to the design of the study. GM and MPF prepared the statistical power analysis. NS, MB, SC and GM will assist in data management. NS will be responsible for recruitment of patients and the acquisition of data from Aberdeen Royal Infirmary and verification and interpretation of data from all sites. SS, KS and SAP will be responsible for the recruitment of patients. SAP will be responsible for the acquisition of data from St Georges Hospital. C J Neil will be responsible for the interpretation of a subset of CMR scans. MPF obtained research funding and has also given a final approval of the version to be published. All authors read and approved the final manuscript.
